# Improvement of renal function after transcatheter aortic valve replacement and its impact on survival

**DOI:** 10.1186/s12882-021-02274-5

**Published:** 2021-03-02

**Authors:** Dominik Kylies, Sandra Freitag-Wolf, Florian Fulisch, Hatim Seoudy, Christian Kuhn, Lars Philipp Kihm, Thomas Pühler, Georg Lutter, Astrid Dempfle, Norbert Frey, Thorsten Feldkamp, Derk Frank

**Affiliations:** 1 Department of Internal Medicine IV, Nephrology and Hypertensiology, University Hospital Schleswig-Holstein, Kiel, Germany; 2 Present Address: Department of Internal Medicine III, Nephrology, Rheumatology and Endocrinology, University Hospital Hamburg (UKE), Hamburg, Germany; 3 Institute of Medical Informatics and Statistics, University Hospital Schleswig-Holstein, Kiel, Germany; 4 Department of Internal Medicine III, Cardiology, Angiology and Critical Care, University Hospital Schleswig-Holstein, Arnold-Heller-Str. 3, 24105 Kiel, Germany; 5 DZHK (German Centre for Cardiovascular Research), Partner Site Hamburg/ Kiel/ Lübeck, Kiel, Germany; 6 Department of Internal Medicine I, University Hospital Heidelberg, Heidelberg, Germany; 7 Department of Cardiovascular Surgery, University Hospital Schleswig-Holstein, Kiel, Germany

**Keywords:** TAVR, Renal function, Renal improvement, Cardiorenal syndrome

## Abstract

**Background:**

Chronic kidney disease as well as acute kidney injury are associated with adverse outcomes after transcatheter aortic valve replacement (TAVR). However, little is known about the prognostic implications of an improvement in renal function after TAVR.

**Methods:**

Renal improvement (RI) was defined as a decrease in postprocedural creatinine in μmol/l of ≥1% compared to its preprocedural baseline value. A propensity score representing the likelihood of RI was calculated to define patient groups which were comparable regarding potential confounders (age, sex, BMI, NYHA classification, STS score, log. EuroSCORE, history of atrial fibrillation/atrial flutter, pulmonary disease, previous stroke, CRP, creatinine, hsTNT and NT-proBNP). The cohort was stratified into 5 quintiles according to this propensity score and the survival time after TAVR was compared within each subgroup.

**Results:**

Patients in quintile 5 (*n* = 93) had the highest likelihood for RI. They were characterized by higher creatinine, lower eGFR, higher NYHA class, higher NT-proBNP, being mostly female and having shorter overall survival time. Within quintile 5, patients without RI had significantly shorter survival compared to patients with RI (*p* = 0.002, HR = 0.32, 95% CI = [0.15–0.69]). There was no survival time difference between patients with and without RI in the whole cohort (*p* = 0.12) and in quintiles 1 to 4 (all *p* > 0.16). Analyses of specific subgroups showed that among patients with NYHA class IV, those with RI also had a significant survival time benefit (*p* < 0.001, HR = 0.15; 95%-CI = [0.05–0.44]) compared to patients without RI.

**Conclusions:**

We here describe a propensity score-derived specific subgroup of patients in which RI after TAVR correlated with a significant survival benefit.

**Supplementary Information:**

The online version contains supplementary material available at 10.1186/s12882-021-02274-5.

## Background

Symptomatic aortic stenosis (AS) is one of the most prevalent forms of valvular heart disease in the elderly [1, 2] with rates of up to 4.6 and 8% at 75 and 85 years of age [2]. It is associated with a high morbidity and mortality if untreated [3, 4].

While surgical aortic valve replacement (SAVR) still represents the standard treatment in low-risk patients, transcatheter aortic valve replacement (TAVR) has emerged as an established alternative in intermediate- and high-risk patients [5].

Several studies have already identified clinical parameters and biomarkers that may improve patient risk stratification (e.g. CK-MB, high-sensitivity Troponin T, BNP and NT-proBNP) [6]. In addition, there are also surgical risk prediction models such as the logistic EuroSCORE (Log ES), its revised and updated version EuroSCORE II, and the Society of Thoracic Surgeons (STS) score [7–10].

Renal function is a parameter of particular interest due to its close relationship with systemic perfusion and cardiac function [11, 12]. It is well-known that preexisting chronic kidney disease (CKD) is associated with adverse outcomes after valve replacement [13] and that acute kidney injury after TAVR is an independent predictor of mortality [14, 15]. Although many studies have investigated the impact of acute kidney injury after TAVR, little is known about the prognostic implications of an improvement in renal function after TAVR. Although some studies have reported an incidence of improvement in kidney function after TAVR in up to 50% of patients [16–18], data regarding the impact of this effect on survival are scarce.

Thus, we here aim to investigate the frequency of an improvement in renal function after TAVR and its prognostic relevance regarding survival.

## Methods

### Study design

Using our TAVR database, we identified a total of 466 patients who underwent transfemoral (TF) TAVR at our institution between March 2009 and June 2016 with a complete data set regarding renal function. Patients with missing relevant data or non-TF access were excluded from our analysis. All patients underwent diagnostic coronary angiography prior to TAVR with percutaneous coronary intervention performed if necessary.

### Data collection

The following baseline patient characteristics were collected before TAVR: age, sex, BMI, NYHA classification, left ventricular ejection fraction, history of atrial flutter/fibrillation, relevant coronary artery disease (CAD), COPD, diabetes mellitus, dyslipidemia, hypertension, peripheral artery disease, cerebrovascular disease, and need for dialysis. We also calculated the STS score as well as log. EuroSCORE and EuroSCORE II. In addition to these clinical parameters, blood samples were taken 1 day before as well as 3 days and 7 days after TAVR for the assessment of complete blood count, serum creatinine, high-sensitive Troponin T, NT-proBNP, urea, and CRP. eGFR was calculated as ml/min/1.73m^2^ using the MDRD formula.

For the majority of patients we did not have creatinine values earlier than day 1. We thus did not pre-define chronic kidney disease. A small subset of patients however required chronic hemodialysis (*n* = 5, 1,1%).

Patient survival was assessed by phone call follow-up by either contacting the patients or their general practitioner and cardiologist. Other than assessment of survival, no other clinical follow-up data (e.g. ECGs) are available. Only follow-up until 24 months after TAVR was considered in this analysis as we were interested in short to medium term effects of renal improvement immediately after TAVR.

### Statistical analysis

Renal improvement (RI) was defined as a decrease in postprocedural creatinine in μmol/l of ≥1% compared to its preprocedural baseline value similar to the definition previously used by Voigtländer et al. [18]. As sensitivity analyses for our main results, we also used a definition of RI based on a decrease in creatinine of ≥5% or ≥ 10% and also defined RI based on an increase in eGFR of either ≥1%, ≥ 5% or ≥ 10%.

Dichotomous and categorical data are presented using percentages, while continuous variables are summarized using median, lower and upper quartile (all relevant variables were not normally distributed). The associations between RI and other clinical variables were assessed by chi-square tests or Wilcoxon rank sum tests, as appropriate. By logistic regression modeling a propensity score for RI [15] was calculated to create patient groups which are comparable regarding possible confounders such as age, sex, BMI, NYHA score, STS score, log. EuroSCORE, atrial fibrillation, COPD, cerebrovascular disease, CRP, creatinine, hsTNT and NT-proBNP (supplementary table 1). A classical 1:1 propensity score matching resulted in unsatisfactory solutions due to difficulties in finding adequate matching pairs, which would drastically reduce the sample size. Thus, we divided our cohort into 5 quintiles according to this renal-specific propensity score (“stratification on the propensity score”), which represents the likelihood of RI. Differences in clinical variables between patient groups defined by propensity score quintiles were assessed by chi-square or Kruskall-Wallis tests.

The survival time for the first 24 months after TAVR was compared between patients with and without RI for the total cohort (using a log-rank test stratified by propensity score quintile) as well as within each quintile (simple log-rank tests). To quantify the effect of RI, the Hazard Ratio (HR) was additionally estimated from a simple Cox regression model. Moreover, further subgroup analyses were performed in an explorative manner: separately for patients with RI and for patients without RI, strata 1–4 were merged and compared with stratum 5 regarding survival time, the effect of RI on survival time was compared for NYHA classes I-IV separately, as well as for patients with high NT-proBNP levels (upper quartile) and those with lower levels (quartile 1–3). As a sensitivity analysis, all pre-procedural prognostic factors, which were significant in the log rank test (*p* < 0.05), were included in a multiple Cox regression model with interaction terms between RI and other relevant variables. Following backward selection was based upon the likelihood ratio criteria. The proportional hazard assumption was checked using weighted residuals and none of the prognostic factors were found to violate this assumption.

All statistical analyses were performed using SPSS version 22 and the statistical software R version 3.2.2 (package survival). In the stratified log-rank test, a *p*-value ≤0.05 was considered to be statistically significant, whereas in the following multiple comparisons the Bonferroni-Holm method was used.

## Results

### Patient characteristics

Patient characteristics are presented in Table 1. The study cohort consisted of a total of 466 Patients with AS who underwent TF-TAVR between the years 2009 and 2016. The median age was 81 years, a total of 200 patients were male (42.9%). Forty-nine patients had an LVEF < 35% (10.5%), the majority of patients (*n* = 328, 70.4%) suffered from severe dyspnea (NYHA classes III and IV). Patients exhibited comorbidities such as coronary artery disease (*n* = 327, 70.2%), arterial hypertension (*n* = 422, 90.6%), history of atrial fibrillation (*n* = 204, 43.8%), dyslipidemia (*n* = 228, 48.9%), and diabetes mellitus (*n* = 141, 30.3%). A large proportion of patients had preexisting chronic kidney disease, as measured by preprocedural creatinine and eGFR. Values for NT-proBNP and hsTNT were also elevated. The median logistic EuroSCORE the EuroSCORE II, and the STS score.

**Table 1:** Baseline patient characteristics

	All Median [Quartile 1 - Quartile 3] or n (%) *N* = 466	Renal improver (RI) Median [Quartile 1 - Quartile 3] or n (%) *N* = 255	Non-Improver (non-RI) Median [Quartile 1 - Quartile 3] or n (%) *N* = 211	*P*-value (RI vs. non-RI)
Age (years)	81 [77–86]	82 [78–87]	81 [77–85]	0.006
Males	200 (42.9)	97 (38.0)	103 (48.8)	0.019
Body-Mass-Index (kg/m^2^)	26.30 [23.44–29.63]	26.01 [23.03–29.14]	26.77 [23.83–30.25]	0.049
LVEF < 35%	49 (10.5)	27 (10.6)	22 (10.4)	0.582
NYHA class III or IV	328 (70.4)	190 (74.5)	138 (65.4)	0.070
Comorbidities				
Coronary artery disease	327 (70.2)	182 (71.4)	145 (68.7)	0.533
Atrial fibrillation	204 (43.8)	117 (45.9)	87 (41.2)	0.314
Hypertension	422 (90.6)	230 (90.2)	192 (91.0)	0.769
Dyslipidemia	228 (48.9)	119 (46.7)	109 (51.7)	0.283
Cerebrovascular disease	84 (18.0)	45 (17.6)	39 (18.5)	0.81
Diabetes mellitus	141 (30.3)	77 (30.2)	64 (30.3)	0.975
Peripheral artery disease	63 (13.5)	31 (12.2)	32 (15.2)	0.344
COPD	60 (12.9)	33 (12.9)	27 (12.8)	0.963
Dialysis	5 (1.1)	3 (1.2)	2 (0.9)	0.812
Logistic EuroSCORE	16.05 [9.69–24.34]	16.35 [10.36–24.40]	15.70 [9.00–24.23]	0.284
EuroSCORE II	4.32 [2.73–7.36]	4.66 [3.04–7.69]	3.92 [2.43–6.77]	0.011
STS score (%)	4.38 [2.86–6.50]	4.43 [3.10–6.52]	4.30 [2.56–6.48]	0.167
NT-proBNP (ng/l)	1816 [714–3928]	2183 [706–4348]	1489 [715–3276]	0.054
hsTNT (pg/ml)	25.40 [15.70–45.55]	25.40 [16.80–50.10]	25.40 [15.00–42.20]	0.298
eGFR (ml/min/1.73m^2^)	54 [37–76]	49 [34–72]	56 [43–78]	0.001
Creatinine (μmol/l)	99.12 [78.41–124.20]	103.55 [79.0–137.0]	96.55 [77.88–118.00]	0.021
Urea (mmol/l)	7.30 [5.55–10.55]	7.70 [5.70–11.49]	6.83 [5.49–9.70]	0.010
CRP (mg/l)	3.7 [1.4–11.1]	4.0 [1.6–11.7]	3.3 [1.4–9.5]	0.431

*LVEF* Left ventricular ejection fraction, *NYHA* New York Heart Association functional class, *COPD* Chronic obstructive pulmonary disease, *STS* Society of Thoracic Surgeons, *NT-proBNP* N-terminal pro-B-type natriuretic peptide, *hsTNT* High-sensitive Troponin T, *eGFR* Estimated glomerulare filtration rate, *CRP* C-reactive protein; *p*-value for qui-square or Wilcoxon rank sum test

### Frequency of renal improvement

According to our criteria described above, RI after TAVR was observed in 255 (54.7%) of a total of 466 patients. Patients with RI were generally slightly older (median 82 vs. 81 years, *p* = 0.006), more frequently female (62% vs. 51.2%, *p* = 0.019) and had slightly lower BMI (median 26.01 vs. 26.77, *p* = 0.049). They also had higher EuroSCORE II (median 4.66 vs. 3.92, *p* = 0.01), lower eGFR (median 49 vs. 57, *p* = 0.001), higher creatinine (median 103.55 μmol/l vs. 96.55 μmol/l, *p* = 0.021), and higher urea (median 7.7 vs. 6.8 mmol/l, *p* = 0.01) at baseline compared to patients without RI (Table 1). Among the subgroup of patients with RI, the median improvement in creatinine was 12, and 75% of the patients had an improvement of at least 6%.

### Description of propensity score quintiles

Detailed patient characteristics for each propensity score quintile are summarized in Table 2. These quintiles reflect the monotonous increase in the likelihood of RI, with a frequency of 36.2% in the lowest quintile to 73.1% in the highest quintile. Patients in higher propensity score strata were older and more often female, had a higher NYHA class, higher initial NT-proBNP, and a higher rate of atrial fibrillation which is generally associated with poorer prognosis. Interestingly, there were fewer patients with diabetes mellitus and dyslipidemia in higher propensity score strata.

**Table 2:** Detailed patient characteristics for each propensity score quintile

	Propensity-Score Quintile 1 n (%) or Median [Quartile 1 - Quartile 3] *N* = 94	Propensity-Score Quintile 2 n (%) or Median [Quartile 1 - Quartile 3] *N* = 93	Propensity-Score Quintile 3 n (%) or Median [Quartile 1 - Quartile 3] *N* = 93	Propensity-Score Quintile 4 n (%) or Median [Quartile 1 - Quartile 3] *N* = 93	Propensity-Score Quintile 5 n (%) or Median [Quartile 1 - Quartile 3] *N* = 93	*P*-value
Renal improvement	34 (36.2)	42 (45.2)	52 (55.9)	59 (63.4)	68 (73.1)	< 0.001
Age (years)	76 [73–79]	81 [77–85]	82 [79–86]	83 [80–87]	86 [81–90]	< 0.001
Males	80 (85.1)	46 (49.5)	32 (34.4)	20 (21.5)	22 (23.7)	< 0.001
Body-Mass-Index (kg/m^2^)	28.34 [25.61–32.25]	26.67 [24.14–30.48]	27.34 [23.83–29.59]	25.15 [22.95–29.35]	23.6 [21.69–26.63]	< 0.001
LVEF < 35%	13 (13.8)	7 (7.5)	10 (10.8)	9 (9.7)	10 (10.8)	0.31
NYHA class III or IV	40 (42.6)	51 (54.8)	64 (68.8)	85 (91.4)	88 (94.6)	< 0.001
Comorbidities						
Coronary artery disease	75 (79.8)	65 (69.9)	65 (69.9)	61 (65.6)	61 (65.6)	0.20
Atrial fibrillation	35 (37.2)	27 (29.0)	40 (43.0)	50 (53.8)	52 (55.9)	0.001
Hypertension	86 (91.5)	87 (93.5)	80 (86)	85 (91.4)	84 (90.3)	0.49
Dyslipidemia	56 (59.6)	56 (60.2)	44 (47.3)	40 (43.0)	32 (34.4)	0.001
Cerebrovascular disease	15 (16.0)	22 (23.7)	20 (21.5)	12 (12.9)	15 (16.1)	0.30
Diabetes mellitus	39 (41.5)	26 (28.0)	38 (40.9)	21 (22.6)	17 (18.3)	0.001
Peripheral artery disease	17 (18.1)	13 (14.0)	12 (12.9)	12 (12.9)	9 (9.7)	0.57
COPD	14 (14.9)	6 (6.5)	10 (10.8)	18 (19.4)	12 (12.9)	0.11
Dialysis	1 (1.1)	0 (0.0)	1 (1.1)	1 (1.1)	2 (2.2)	0.73
Logistic EuroSCORE	12.8 [7.9–21.7]	14.28 [8.37–24.2]	12.91 [9.25–23.52]	15.08 [10.3–25.18]	19.67 [14.12–27.85]	< 0.001
EuroSCORE II	4.1 [1.95–7.63]	3.54 [2.38–7.07]	3.86 [2.67–7.06]	4.29 [2.94–7.01]	5.48 [3.74–8.02]	0.001
STS score	3.25 [1.99–5.54]	3.73 [2.4–6.07]	4.23 [3–5.82]	4.73 [3.27–5.93]	6.29 [4.31–8.46]	< 0.001
NT-proBNP (ng/l)	1097 [474–2194]	1016 [541–2500]	1130 [693–3092]	2184 [907–3802]	7042 [2969–12,890]	< 0.001
hsTNT (pg/ml)	24.7 [15.25–43.83]	23.3 [14.05–37]	21.8 [13.45–39.4]	23.3 [16.45–44.1]	38 [23.7–72.65]	< 0.001
eGFR (ml/min/1.73m^2^)	73 [52–98]	64 [42–85]	52 [42–69]	50 [35–66]	36 [26–49]	< 0.001
Creatinine (μmol/l)	98.24 [76.59–117.62]	91.51 [75.62–119.5]	93.81 [77–117.41]	98.24 [77–120.98]	120 [95.41–165.94]	< 0.001
Urea (mmol/l)	6.66 [5.38–9.49]	6.5 [5.2–10.05]	7.33 [5.33–9.68]	7.16 [5.7–10.05]	9.82 [6.23–13.64]	< 0.001
CRP (mg/l)	3.2 [1.2–7.7]	2.3 [1.1–5.6]	3.1 [1.5–8.3]	4.9 [2.05–12.1]	6.4 [1.8–24.5]	< 0.001

*LVEF* Left ventricular ejection fraction, *NYHA* New York Heart Association functional class, *COPD* Chronic obstructive pulmonary disease, *STS* Society of Thoracic Surgeons, *NT-proBNP* N-terminal pro-B-type natriuretic peptide, *hsTNT* High-sensive Troponin T, *eGFR* Estimated glomerulare filtration rate, *CRP* C-reactive protein; *p*-value for Kruskall-Wallis test between propensity score quintiles

### Association of RI with survival

**Fig. 1:**
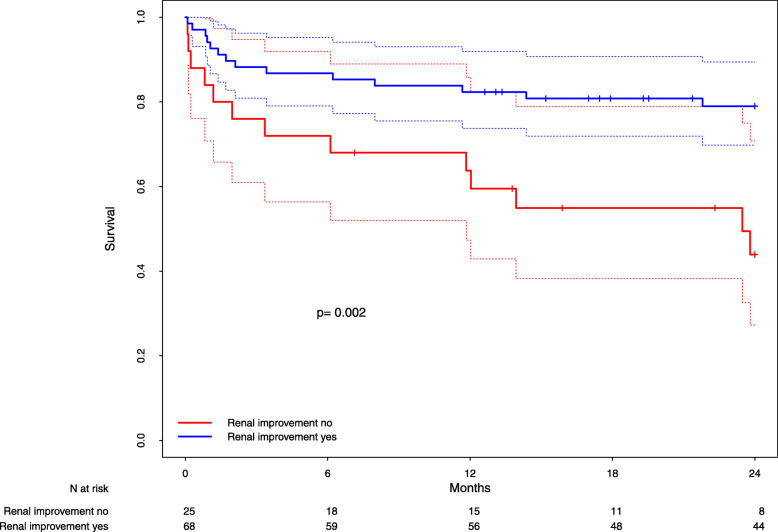
Survival and renal improvement (propensity stratum 5). Kaplan-Meier-Estimates of survival in patients with (blue line) and without (red line) renal improvement in propensity stratum 5. Dottet lines: 95% confidence bands

In the total cohort, survival time did not differ between patients with or without RI (adjusted for propensity score strata, *p* = 0.13). However, there was a statistically significant benefit in the survival time of patients with RI (vs. those without RI) in propensity score stratum 5 (Fig. 1, *p* = 0.002, HR = 0.32, 95% CI = [0.15–0.69]), whereas no significant difference in the survival time could be observed for patients with RI vs. those without RI in the individual propensity score strata 1–4 (supplementary figures 1, 2, 3 and 4; *p* > 0.17 for all strata). The same risk group was identified in the complementary comparisons: in the subgroup of patients without RI, there was a significantly decreased survival time in propensity score stratum 5 compared to strata 1–4 (*p* < 0.001, HR = 2.93, 95% CI = [1.58–5.46], supplementary figure 5), whereas there was no significant difference among patients with RI (supplementary figure 6). Thus, only one group of patients had a markedly poorer prognosis than all others: those in stratum 5 who did not achieve RI had an estimated two-year survival rate of only 43.9%, while patients in all other subgroups (stratum 5 with RI and strata 1 to 4 irrespective of RI) had estimated two-year survival rates of 75.7% (between 64.8 and 85.5% in individual subgroups, see supplementary figure 7).

**Fig. 2:**
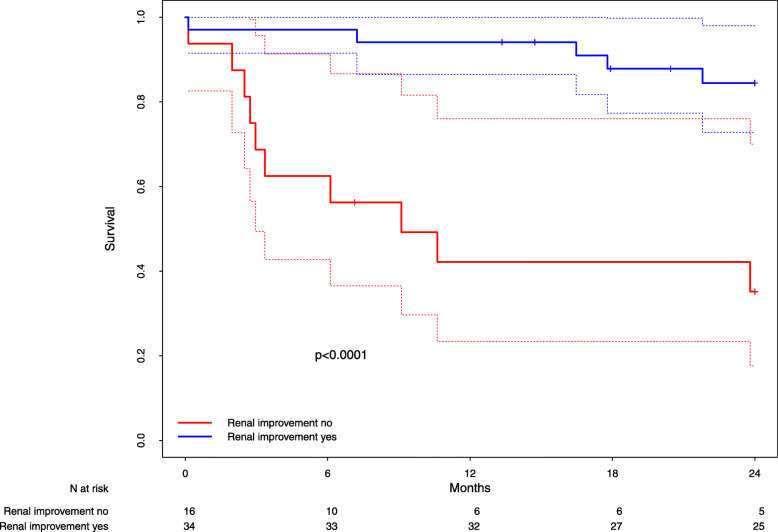
Survival of patients with RI vs. patients without RI among the subgroup of patients with NYHA IV. Kaplan-Meier-Estimates of survival in patients with (blue line) and without (red line) renal improvement among the subgroup of patients with NYHA IV. Dottet lines: 95% confidence bands

Interestingly, in the highest NYHA class (IV; *n* = 50 patients; of these, 27 were in propensity score stratum 5), RI was associated with a significant increase in survival time (*p* < 0.001, Fig. 2, HR = 0.15; 95%-CI = [0.05–0.44]), whereas patients with RI in NYHA classes II and III showed no statistically significant improvement in survival time compared to patients without RI (*p* > 0.8, supplementary figures 8 & 9).

**Fig. 3:**
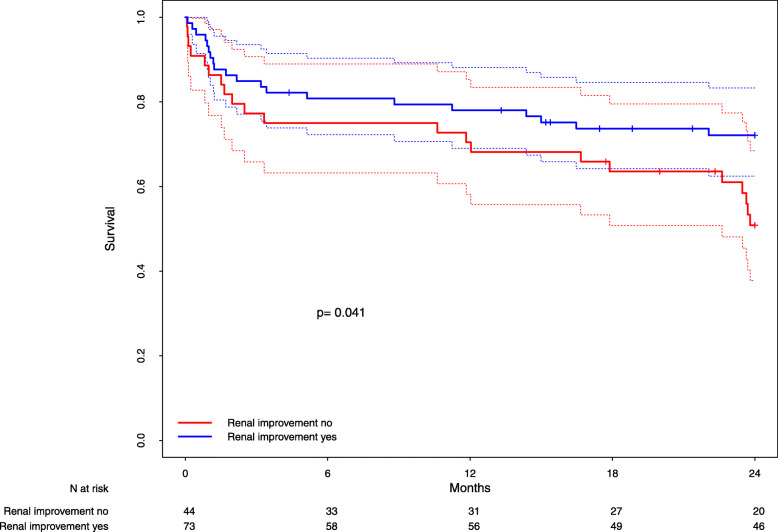
Survival of patients with RI vs. patients without RI for the upper quartile of NT-proBNP. Kaplan-Meier-Estimates of survival in patients with (blue line) and without (red line) renal improvement among the subgroup of patients with baseline NT-proBNP values in the upper quartile. Dottet lines: 95% confidence bands

Similarly, patients in the highest quartile for NT-proBNP with RI showed a significantly longer survival compared to patients without RI (*p* = 0.04, Fig. 3, HR = 0.53; 95%-CI = [0.29–0.98]), whereas patients in the lower quartiles 1–3 for NT-proBNP showed no difference in survival depending on RI (*p* = 0.87, supplementary figure 10).

In our first sensitivity analysis, the Cox regression model showed a significant effect of RI (*p* = 0.02) when interaction terms between RI and NYHA as well as between RI and NT-proBNP (NYHA*RI: *p* = 0.04, NT-proBNP*RI: *p* = 0.0015; supplementary table 2) were included in the model. Further sensitivity analyses confirmed the robustness of the survival benefit of patients with RI in propensity stratum 5 and in the subgroup of patients with NYHA class IV, using different definitions of “RI” based on larger improvements (5% or 10%) of either serum creatinine or eGFR with similar estimated HRs (see supplementary table 3). The survival benefit in the subgroup of patients with elevated NT-proBNP (baseline values in the upper quartile) was not as clear in the sensitivity analyses.

## Discussion

About half of all patients experience renal improvement immediately after TAVR in our study. For most patients, this does not have a clear effect on survival. However, in one specific subgroup, survival was markedly decreased if patients did not achieve renal improvement. These patients in quintile 5 of our propensity score, which represents the highest likelihood for RI, can be characterized as being mostly female, having poor initial renal function (high creatinine and low eGFR), significant comorbidities and more severe symptoms (higher NYHA class, higher STS score, higher initial NT-proBNP), and having shorter overall-survival time. RI in this subgroup increases survival to that of patients in lower propensity score strata (i.e. with better initial prognosis).

Propensity score methods are common approaches to minimize the effects of confounding when estimating the effects of a potential risk factor in an observational study. We used stratification on the propensity score such that five equally sized groups were analyzed conceptually both as a meta-analysis and for each stratum individually. It was demonstrated that stratifying on the quintiles of a continuous confounding variable eliminates approximately 90% of the bias due to that variable [15]. The factors used in this score were clinical and laboratory parameters for the assessment of cardiac and renal function as well as relevant cardiovascular comorbidities and established risk-scores.

There are several possible explanations why patients in propensity score stratum 5 benefitted most from RI despite the higher morbidity compared to patients in lower propensity score strata. One aspect might be that these patients had the most severe pre-existing chronic kidney disease and thus improvement had the highest impact in this group. Another factor could be that improved hemodynamics after TAVR leads to a better kidney perfusion and thus addresses an underlying functional deficit that is potentially reversible as opposed to a more structural kidney damage.In addition to that, an improvement in kidney function after TAVR could also be due to recovery of a previously existing acute kidney injury. Due to the study design those factors cannot be distinguished with certainty.

Interestingly, the BMI was higher and the rates of dyslipidemia and diabetes mellitus were significantly lower in the propensity score stratum 5. These factors by themselves are well established risk-factors for the development of a structural chronic kidney disease (e.g., diabetic nephropathy) which would not respond as well to improved hemodynamics as a functional impairment alone. It is well established that chronic kidney disease is linked to an exponentially increased absolute risk for mortality with decreasing renal function as shown in a meta-analysis from 2006 [19].

Another hypothesis could be that not all patients benefit from TAVR in terms of improved hemodynamics. Voigtländer et al. [18] showed an improvement of cardiac output in patients with an increase in eGFR after TAVR, but not in patients with a decline in eGFR. In addition to that, an association between renal perfusion index and cardiac left ventricular systolic function has been previously shown [20].

Moreover, both renal and cardiac fibrosis are promoted in the setting of chronic cardiorenal syndrome via multiple pathways [21]; improvements in hemodynamics after TAVR could thus slow down the progression of a structural chronic kidney disease.

In propensity score strata 1–4, we also detected improved survival in patients without RI when comparing the data to stratum 5. This finding is not surprising. As described above, the propensity score for RI was calculated to create patient groups which are comparable regarding potential confounders. As a result of this propensity scoring, patients in higher strata not only have a better chance for RI, they also have higher rates of factors for adverse outcomes such as higher age, higher NYHA class and higher initial NT-proBNP. What is intriguing about this finding is that the decreased survival in patients was only seen in patients without RI. Patients with RI had similar, much higher survival regardless of the propensity score stratum. RI could thus define a lower risk subset of patients.

Of note, in highly symptomatic patients (belonging to NYHA class IV), renal improvement was associated with significantly improved survival, in fact this group showed the greatest benefit. There are various possible explanations to this specific finding. In addition to the aspects discussed above, patients with higher stages of chronic heart failure are dependent on their heart failure medication to improve morbidity and mortality. Recent studies have shown that lower eGFR was associated with higher rates of adverse reactions of heart failure medication that lead to drug discontinuation [22]. The improved survival in patients with NYHA class IV and RI could thus at least in parts be explained by higher rates of drug adherence. Further studies will be needed to address this finding.

## Conclusions

Taken together, in a subgroup of patients with a high propensity score reflecting a high probability of renal improvement, not achieving renal improvement after TAVR was associated with significantly worse long-term survival. Further subgroup analyses in highly symptomatic patients (NYHA class IV) and patients with elevated heart failure biomarkers (upper quartile of NT-proBNP) further confirm the relevance of renal improvement for long-term survival in high-risk patients.

## Supplementary Information

**Additional file 1: Supplementary table 1.** Variables included in the propensity score with their ORs from coefficients of multiple logistic regression model with outcome renal improvement. Fitted values of this logistic model were used to calculate the propensity score for each patient. **Supplementary table 2.** Multiple Cox model of two-year survival after TAVI. **Supplementary table 3.** Sensitivity analyses using different definitions of RI, based on improvement thresholds of serum creatinine or eGFR.

**Additional file 2: Supplementary figure 1.** Kaplan-Meier-Estimates of survival in patients with (blue line) and without (red line) renal improvement in propensity stratum 1. **Supplementary figure 2.** Kaplan-Meier-Estimates of survival in patients with (blue line) and without (red line) renal improvement in propensity stratum 2. **Supplementary figure 3.** Kaplan-Meier-Estimates of survival in patients with (blue line) and without (red line) renal improvement in propensity stratum 3. **Supplementary figure 4.** Kaplan-Meier-Estimates of survival in patients with (blue line) and without (red line) renal improvement in propensity stratum 4. **Supplementary figure 5.** Kaplan-Meier-Estimates of survival in patients in propensity strata 1 to 4 (blue line) and in propensity stratum 5 (red line) among the subgroup of patients without renal improvement. **Supplementary figure 6.** Kaplan-Meier-Estimates of survival in patients in propensity strata 1 to 4 (blue line) and in propensity stratum 5 (red line) among the subgroup of patients with renal improvement. **Supplementary figure 7.** Kaplan-Meier-Estimates of survival in patients with (dashed lines) and without (solid lines) renal improvement, separately for all 5 propensity strata (stratum 1: black, stratum 2: green, stratum 3: blue, stratum 4: grey, stratum 5: red). **Supplementary figure 8.** Kaplan-Meier-Estimates of survival in patients with (blue line) and without (red line) renal improvement among the subgroup of patients with NYHA II. **Supplementary figure 9.** Kaplan-Meier-Estimates of survival in patients with (blue line) and without (red line) renal improvement among the subgroup of patients with NYHA III. **Supplementary figure 10.** Kaplan-Meier-Estimates of survival in patients with (blue line) and without (red line) renal improvement among the subgroup of patients with baseline NT-proBNP values in the first to third quartile.

## Data Availability

All data analysed during this study are included in this published article (and its supplementary information files).

## Abbreviations

AS: Aortic stenosis
BMI: Body mass index
CI: Confidence intervals
COPD: Chronic obstructive pulmonary disease
eGFR: Estimated glomerular filtration rate
HR: Hazard ratio
hsTNT: High-sensitivity Troponin T
IQR: Interquartile range
LVEF: Left ventricular ejection fraction
NT-proBNP: N-terminal pro-B-type natriuretic peptide
NYHA: New York Heart Association functional class
TAVR: Transcatheter aortic valve replacement
TF: Transfemoral
VARC-2: Valve Academic Research Consortium-2
