# Supplementary material for: Improvement of renal function after transcatheter aortic valve replacement and its impact on survival
Source: BMC Nephrol. 2021 Mar 2;22:77. doi: 10.1186/s12882-021-02274-5 (PMC7923662; doi:10.1186/s12882-021-02274-5)
Supplement: Supplementary file 1 — Additional file 1: Supplementary table 1. Variables included in the propensity score with their ORs from coefficients of multiple logistic regression model with outcome renal improvement. Fitted values of this logistic model were used to calculate the propensity score for each patient. Supplementary table 2. Multiple Cox model of two-year survival after TAVI. Supplementary table 3. Sensitivity analyses using different definitions of RI, based on improvement thresholds of serum creatinine or eGFR. [file 12882_2021_2274_MOESM1_ESM.docx]

|  | OR | 2.50% | 97.50% | p |
| --- | --- | --- | --- | --- |
| Intercept | 0.025 | -7.2 | -0.227 | 0.038 |
| age | 1.048 | 0.01 | 0.084 | 0.014 |
| male sex | 0.588 | -0.948 | -0.121 | 0.012 |
| BMI | 0.971 | -0.069 | 0.01 | 0.149 |
| NYHA class III or IV | 1.353 | 0.006 | 0.604 | 0.047 |
| STS | 0.933 | -0.161 | 0.02 | 0.13 |
| log ES | 0.997 | -0.025 | 0.019 | 0.785 |
| atrial fibrillation | 1.192 | -0.221 | 0.574 | 0.386 |
| COPD | 1.202 | -0.425 | 0.806 | 0.556 |
| previous stroke | 0.948 | -0.564 | 0.46 | 0.837 |
| CRP | 1.005 | -0.005 | 0.017 | 0.351 |
| creatinine | 1.004 | 0.001 | 0.008 | 0.036 |
| hsTNT | 0.998 | -0.01 | 0.006 | 0.58 |
| NT-proBNP | 1.000045 | 0.99998 | 1.00011 | 0.158 |

Supplementary table 1: Variables included in the propensity score with their ORs from coefficients of multiple logistic regression model with outcome renal improvement. Fitted values of this logistic model were used to calculate the propensity score for each patient.

Supplementary table 2: Multiple Cox model of two-year survival after TAVI

|  | HR | 95% CI | | se(coef) | p |
| --- | --- | --- | --- | --- | --- |
| RI | 6.761 | 1.328 | 34.426 | 0.831 | 0.021 |
| age | 1.031 | 1.002 | 1.062 | 0.015 | 0.037 |
| NYHA class III or IV | 1.464 | 0.964 | 2.222 | 0.213 | 0.074 |
| atrial fibrillation | 1.919 | 1.306 | 2.819 | 0.196 | 0.001 |
| COPD | 2.396 | 1.531 | 3.749 | 0.228 | <0.001 |
| hsTNT | 1.011 | 1.004 | 1.017 | 0.003 | 0.001 |
| NT-proBNP | 1.000 | 1.000 | 1.000 | 0.000 | 0.001 |
| RI*NYHA (III or IV) | 0.558 | 0.318 | 0.979 | 0.287 | 0.042 |
| RI*NT-proBNP | 1.000 | 1.000 | 1.000 | 0.000 | 0.002 |

Supplementary table 3: Sensitivity analyses using different definitions of RI , based on improvement thresholds of serum creatinine or eGFR

| Sensitivity analysis 1: only within quintile 5 of propensity score | | | | | |  |
| --- | --- | --- | --- | --- | --- | --- |
|  | n (RI) | n (no RI) | HR | lower 95% CI | upper 05% CI | p-value |
| creatinine 1% | 68 | 25 | 0.32 | 0.15 | 0.69 | 0.002 |
| creatinine 5% | 58 | 35 | 0.66 | 0.33 | 1.31 | 0.20 |
| creatinine 10% | 45 | 48 | 0.56 | 0.28 | 1.17 | 0.12 |
| eGFR 1% | 67 | 26 | 0.45 | 0.20 | 0.98 | 0.05 |
| eGFR 5% | 67 | 26 | 0.22 | 0.07 | 0.65 | 0.006 |
| eGFR 10% | 54 | 39 | 0.60 | 0.28 | 1.29 | 0.19 |
|  |  |  |  |  |  |  |
| Sensitivity analysis 2: only within NYHA IV | | | | |  |  |
|  | n (RI) | n (no RI) | HR | lower 95% CI | upper 05% CI | p-value |
| creatinine 1% | 34 | 16 | 0.15 | 0.05 | 0.44 | <0.001 |
| creatinine 5% | 29 | 21 | 0.27 | 0.09 | 0.79 | 0.02 |
| creatinine 10% | 22 | 28 | 0.26 | 0.07 | 0.91 | 0.03 |
| eGFR 1% | 34 | 16 | 0.15 | 0.05 | 0.44 | 0.001 |
| eGFR 5% | 31 | 19 | 0.22 | 0.07 | 0.65 | 0.003 |
| eGFR 10% | 28 | 22 | 0.30 | 0.10 | 0.88 | 0.03 |
|  |  |  |  |  |  |  |
| Sensitivity analysis 3: only within 3rd quartile of NT-proBNP | | | | | |  |
|  | n (RI) | n (no RI) | HR | lower 95% CI | upper 05% CI | p-value |
| creatinine 1% | 73 | 44 | 0.53 | 0.29 | 0.98 | 0.04 |
| creatinine 5% | 64 | 53 | 0.70 | 0.38 | 1.30 | 0.26 |
| creatinine 10% | 48 | 69 | 0.79 | 0.42 | 1.51 | 0.48 |
| eGFR 1% | 73 | 44 | 0.50 | 0.28 | 0.98 | 0.04 |
| eGFR 5% | 68 | 49 | 0.66 | 0.36 | 1.23 | 0.20 |
| eGFR 10% | 58 | 59 | 0.68 | 0.33 | 1.27 | 0.24 |
