# Supplementary material for: Improvement of renal function after transcatheter aortic valve replacement and its impact on survival
Source: BMC Nephrol. 2021 Mar 2;22:77. doi: 10.1186/s12882-021-02274-5 (PMC7923662; doi:10.1186/s12882-021-02274-5)
Supplement: Supplementary file 2 — Additional file 2: Supplementary figure 1. Kaplan-Meier-Estimates of survival in patients with (blue line) and without (red line) renal improvement in propensity stratum 1. Supplementary figure 2. Kaplan-Meier-Estimates of survival in patients with (blue line) and without (red line) renal improvement in propensity stratum 2. Supplementary figure 3. Kaplan-Meier-Estimates of survival in patients with (blue line) and without (red line) renal improvement in propensity stratum 3. Supplementary figure 4. Kaplan-Meier-Estimates of survival in patients with (blue line) and without (red line) renal improvement in propensity stratum 4. Supplementary figure 5. Kaplan-Meier-Estimates of survival in patients in propensity strata 1 to 4 (blue line) and in propensity stratum 5 (red line) among the subgroup of patients without renal improvement. Supplementary figure 6. Kaplan-Meier-Estimates of survival in patients in propensity strata 1 to 4 (blue line) and in propensity stratum 5 (red line) among the subgroup of patients with renal improvement. Supplementary figure 7. Kaplan-Meier-Estimates of survival in patients with (dashed lines) and without (solid lines) renal improvement, separately for all 5 propensity strata (stratum 1: black, stratum 2: green, stratum 3: blue, stratum 4: grey, stratum 5: red). Supplementary figure 8. Kaplan-Meier-Estimates of survival in patients with (blue line) and without (red line) renal improvement among the subgroup of patients with NYHA II. Supplementary figure 9. Kaplan-Meier-Estimates of survival in patients with (blue line) and without (red line) renal improvement among the subgroup of patients with NYHA III. Supplementary figure 10. Kaplan-Meier-Estimates of survival in patients with (blue line) and without (red line) renal improvement among the subgroup of patients with baseline NT-proBNP values in the first to third quartile. [file 12882_2021_2274_MOESM2_ESM.zip › Supp2_RI_S2R3.pdf]

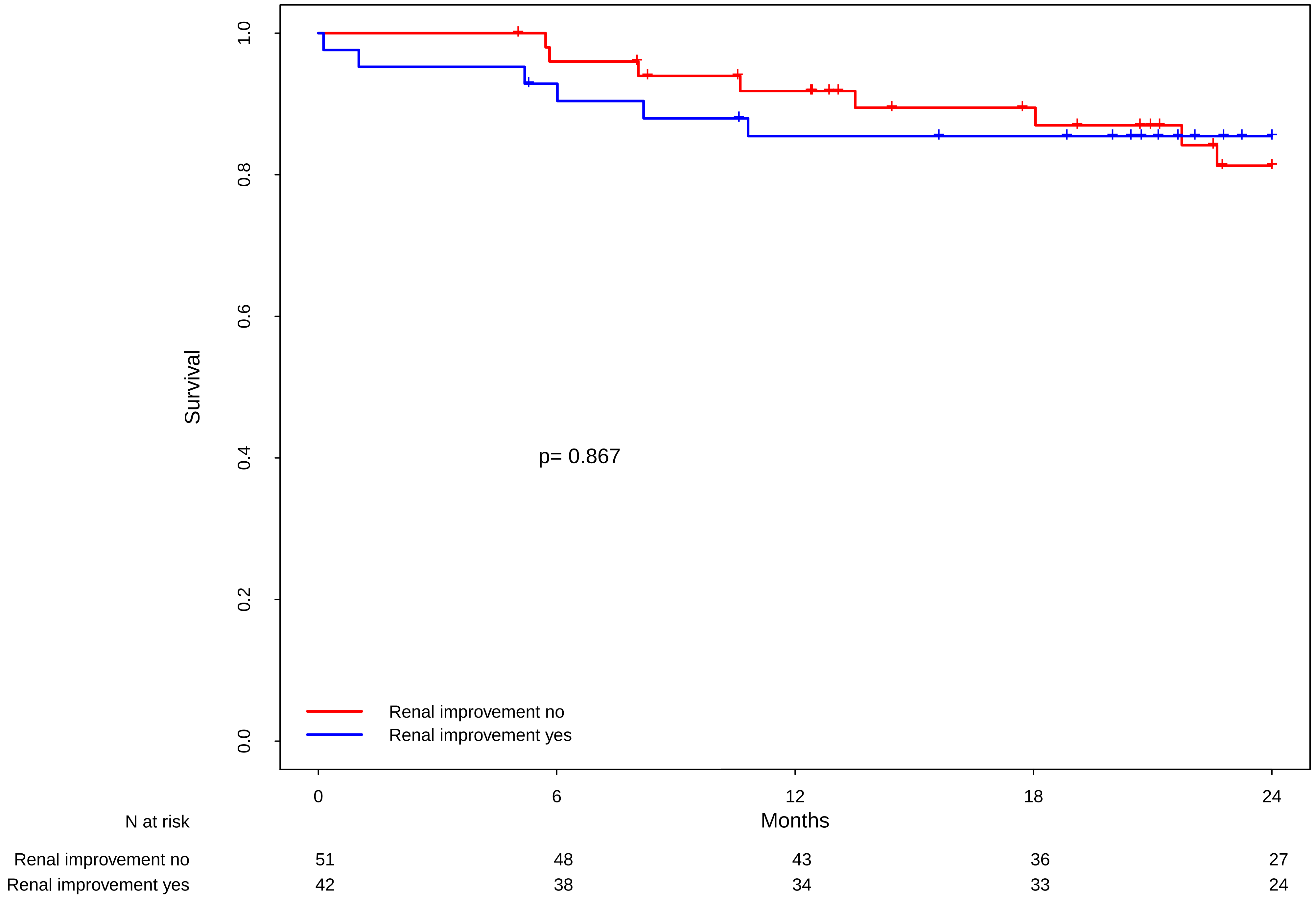

Survival
1.0
0.8
0.6
0.4
0.2
0.0
p= 0.867
Renal improvement no
Renal improvement yes
0
6
12
18
24
Months
N at risk
Renal improvement no 51 48 43 36 27
Renal improvement yes 42 38 34 33 24
