# Supplementary figures and images for: Improvement of renal function after transcatheter aortic valve replacement and its impact on survival
Source: BMC Nephrol. 2021 Mar 2;22:77. doi: 10.1186/s12882-021-02274-5 (PMC7923662; doi:10.1186/s12882-021-02274-5)

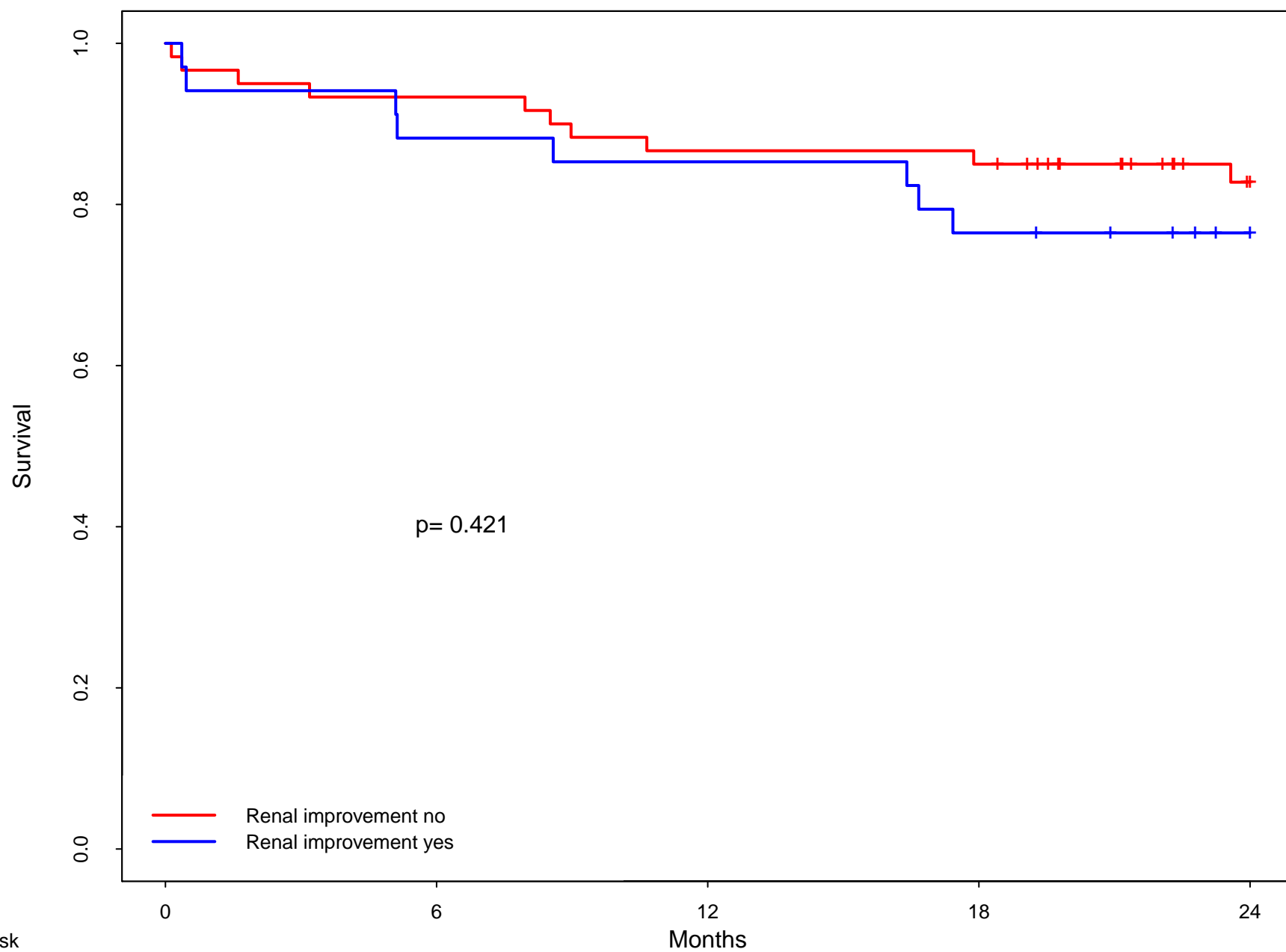

$p=0.421$

Renal improvement no  
Renal improvement yes

N at risk

|    |    |    |    |    |
|----|----|----|----|----|
| 0  | 6  | 12 | 18 | 24 |
| 60 | 56 | 52 | 51 | 36 |
| 34 | 30 | 29 | 26 | 19 |

Supplement: Supplementary file 2 — Additional file 2: Supplementary figure 1. Kaplan-Meier-Estimates of survival in patients with (blue line) and without (red line) renal improvement in propensity stratum 1. Supplementary figure 2. Kaplan-Meier-Estimates of survival in patients with (blue line) and without (red line) renal improvement in propensity stratum 2. Supplementary figure 3. Kaplan-Meier-Estimates of survival in patients with (blue line) and without (red line) renal improvement in propensity stratum 3. Supplementary figure 4. Kaplan-Meier-Estimates of survival in patients with (blue line) and without (red line) renal improvement in propensity stratum 4. Supplementary figure 5. Kaplan-Meier-Estimates of survival in patients in propensity strata 1 to 4 (blue line) and in propensity stratum 5 (red line) among the subgroup of patients without renal improvement. Supplementary figure 6. Kaplan-Meier-Estimates of survival in patients in propensity strata 1 to 4 (blue line) and in propensity stratum 5 (red line) among the subgroup of patients with renal improvement. Supplementary figure 7. Kaplan-Meier-Estimates of survival in patients with (dashed lines) and without (solid lines) renal improvement, separately for all 5 propensity strata (stratum 1: black, stratum 2: green, stratum 3: blue, stratum 4: grey, stratum 5: red). Supplementary figure 8. Kaplan-Meier-Estimates of survival in patients with (blue line) and without (red line) renal improvement among the subgroup of patients with NYHA II. Supplementary figure 9. Kaplan-Meier-Estimates of survival in patients with (blue line) and without (red line) renal improvement among the subgroup of patients with NYHA III. Supplementary figure 10. Kaplan-Meier-Estimates of survival in patients with (blue line) and without (red line) renal improvement among the subgroup of patients with baseline NT-proBNP values in the first to third quartile. [file 12882_2021_2274_MOESM2_ESM.zip › Supp1_RI_S1R3.pdf]

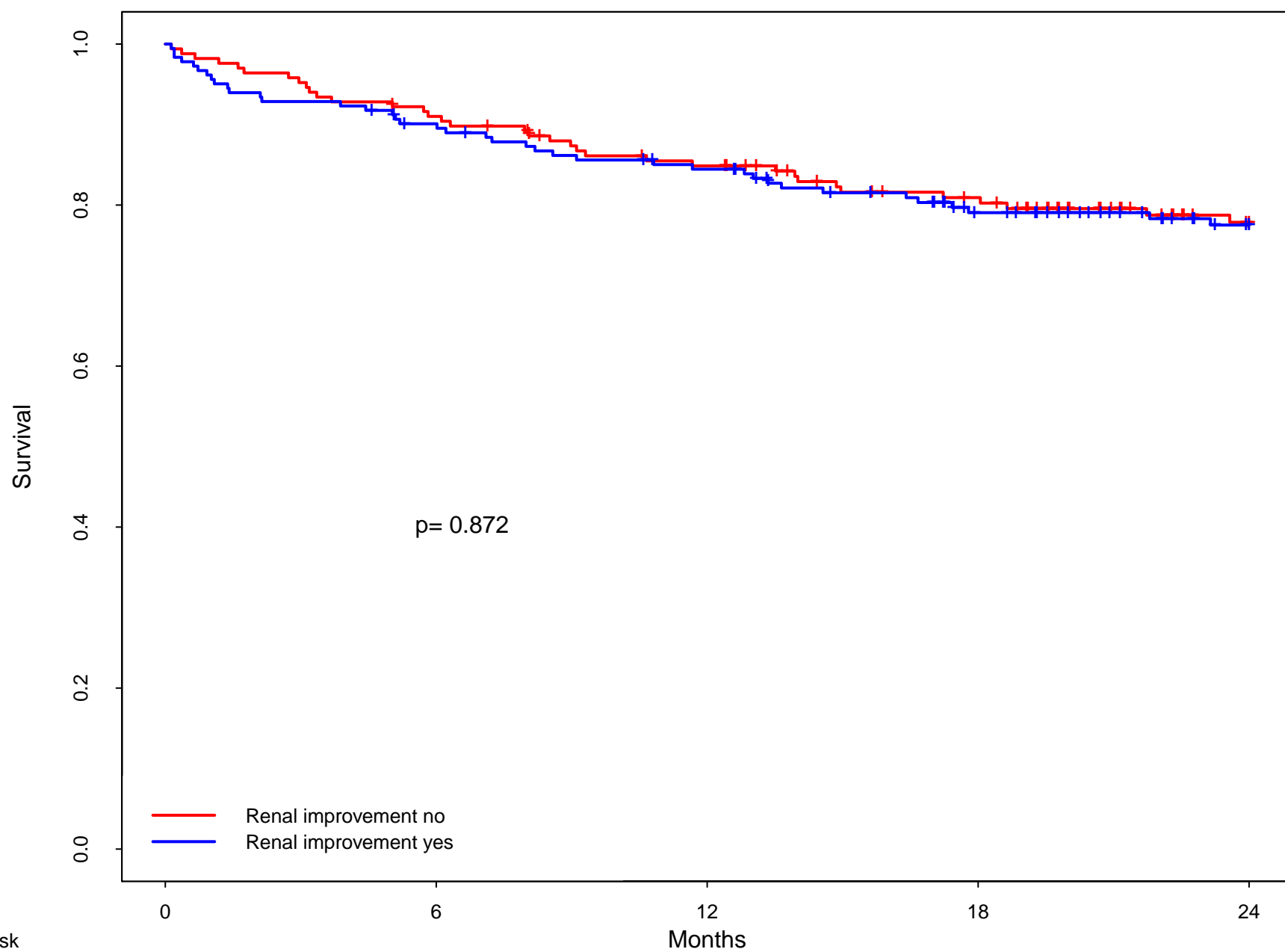

N at risk

|                       |     |     |     |     |    |
|-----------------------|-----|-----|-----|-----|----|
| Renal improvement no  | 167 | 151 | 136 | 119 | 87 |
| Renal improvement yes | 182 | 161 | 148 | 122 | 94 |

Supplement: Supplementary file 2 — Additional file 2: Supplementary figure 1. Kaplan-Meier-Estimates of survival in patients with (blue line) and without (red line) renal improvement in propensity stratum 1. Supplementary figure 2. Kaplan-Meier-Estimates of survival in patients with (blue line) and without (red line) renal improvement in propensity stratum 2. Supplementary figure 3. Kaplan-Meier-Estimates of survival in patients with (blue line) and without (red line) renal improvement in propensity stratum 3. Supplementary figure 4. Kaplan-Meier-Estimates of survival in patients with (blue line) and without (red line) renal improvement in propensity stratum 4. Supplementary figure 5. Kaplan-Meier-Estimates of survival in patients in propensity strata 1 to 4 (blue line) and in propensity stratum 5 (red line) among the subgroup of patients without renal improvement. Supplementary figure 6. Kaplan-Meier-Estimates of survival in patients in propensity strata 1 to 4 (blue line) and in propensity stratum 5 (red line) among the subgroup of patients with renal improvement. Supplementary figure 7. Kaplan-Meier-Estimates of survival in patients with (dashed lines) and without (solid lines) renal improvement, separately for all 5 propensity strata (stratum 1: black, stratum 2: green, stratum 3: blue, stratum 4: grey, stratum 5: red). Supplementary figure 8. Kaplan-Meier-Estimates of survival in patients with (blue line) and without (red line) renal improvement among the subgroup of patients with NYHA II. Supplementary figure 9. Kaplan-Meier-Estimates of survival in patients with (blue line) and without (red line) renal improvement among the subgroup of patients with NYHA III. Supplementary figure 10. Kaplan-Meier-Estimates of survival in patients with (blue line) and without (red line) renal improvement among the subgroup of patients with baseline NT-proBNP values in the first to third quartile. [file 12882_2021_2274_MOESM2_ESM.zip › Supp10_Surv_RI_in_proBNP1to3R3.pdf]

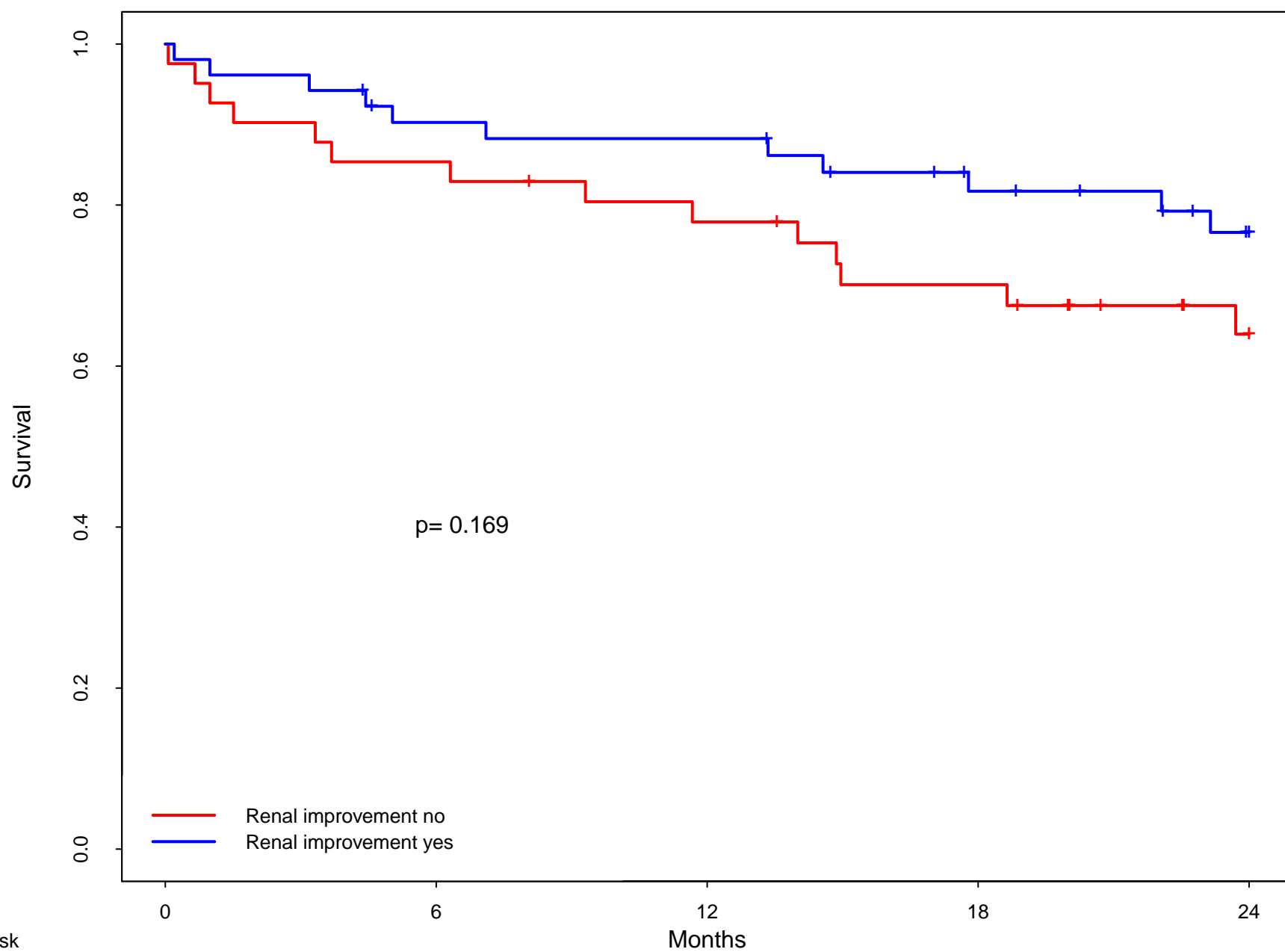

Supplement: Supplementary file 2 — Additional file 2: Supplementary figure 1. Kaplan-Meier-Estimates of survival in patients with (blue line) and without (red line) renal improvement in propensity stratum 1. Supplementary figure 2. Kaplan-Meier-Estimates of survival in patients with (blue line) and without (red line) renal improvement in propensity stratum 2. Supplementary figure 3. Kaplan-Meier-Estimates of survival in patients with (blue line) and without (red line) renal improvement in propensity stratum 3. Supplementary figure 4. Kaplan-Meier-Estimates of survival in patients with (blue line) and without (red line) renal improvement in propensity stratum 4. Supplementary figure 5. Kaplan-Meier-Estimates of survival in patients in propensity strata 1 to 4 (blue line) and in propensity stratum 5 (red line) among the subgroup of patients without renal improvement. Supplementary figure 6. Kaplan-Meier-Estimates of survival in patients in propensity strata 1 to 4 (blue line) and in propensity stratum 5 (red line) among the subgroup of patients with renal improvement. Supplementary figure 7. Kaplan-Meier-Estimates of survival in patients with (dashed lines) and without (solid lines) renal improvement, separately for all 5 propensity strata (stratum 1: black, stratum 2: green, stratum 3: blue, stratum 4: grey, stratum 5: red). Supplementary figure 8. Kaplan-Meier-Estimates of survival in patients with (blue line) and without (red line) renal improvement among the subgroup of patients with NYHA II. Supplementary figure 9. Kaplan-Meier-Estimates of survival in patients with (blue line) and without (red line) renal improvement among the subgroup of patients with NYHA III. Supplementary figure 10. Kaplan-Meier-Estimates of survival in patients with (blue line) and without (red line) renal improvement among the subgroup of patients with baseline NT-proBNP values in the first to third quartile. [file 12882_2021_2274_MOESM2_ESM.zip › Supp3_RI_S3R3.pdf]

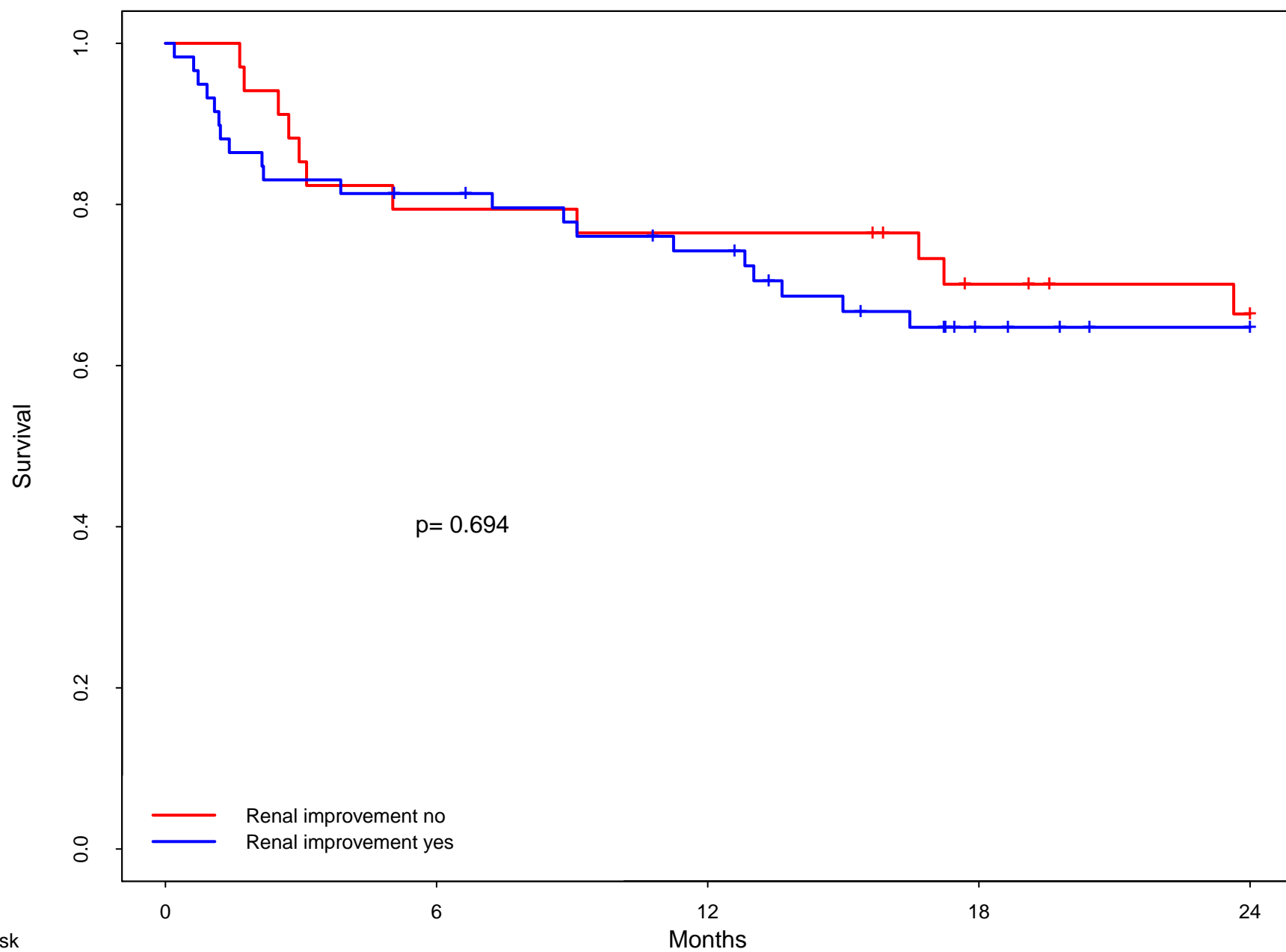

N at risk

Renal improvement no

Renal improvement yes

0 6 12 18 24

34 27 26 21 18

59 47 41 29 25

Supplement: Supplementary file 2 — Additional file 2: Supplementary figure 1. Kaplan-Meier-Estimates of survival in patients with (blue line) and without (red line) renal improvement in propensity stratum 1. Supplementary figure 2. Kaplan-Meier-Estimates of survival in patients with (blue line) and without (red line) renal improvement in propensity stratum 2. Supplementary figure 3. Kaplan-Meier-Estimates of survival in patients with (blue line) and without (red line) renal improvement in propensity stratum 3. Supplementary figure 4. Kaplan-Meier-Estimates of survival in patients with (blue line) and without (red line) renal improvement in propensity stratum 4. Supplementary figure 5. Kaplan-Meier-Estimates of survival in patients in propensity strata 1 to 4 (blue line) and in propensity stratum 5 (red line) among the subgroup of patients without renal improvement. Supplementary figure 6. Kaplan-Meier-Estimates of survival in patients in propensity strata 1 to 4 (blue line) and in propensity stratum 5 (red line) among the subgroup of patients with renal improvement. Supplementary figure 7. Kaplan-Meier-Estimates of survival in patients with (dashed lines) and without (solid lines) renal improvement, separately for all 5 propensity strata (stratum 1: black, stratum 2: green, stratum 3: blue, stratum 4: grey, stratum 5: red). Supplementary figure 8. Kaplan-Meier-Estimates of survival in patients with (blue line) and without (red line) renal improvement among the subgroup of patients with NYHA II. Supplementary figure 9. Kaplan-Meier-Estimates of survival in patients with (blue line) and without (red line) renal improvement among the subgroup of patients with NYHA III. Supplementary figure 10. Kaplan-Meier-Estimates of survival in patients with (blue line) and without (red line) renal improvement among the subgroup of patients with baseline NT-proBNP values in the first to third quartile. [file 12882_2021_2274_MOESM2_ESM.zip › Supp4_RI_S4R3.pdf]

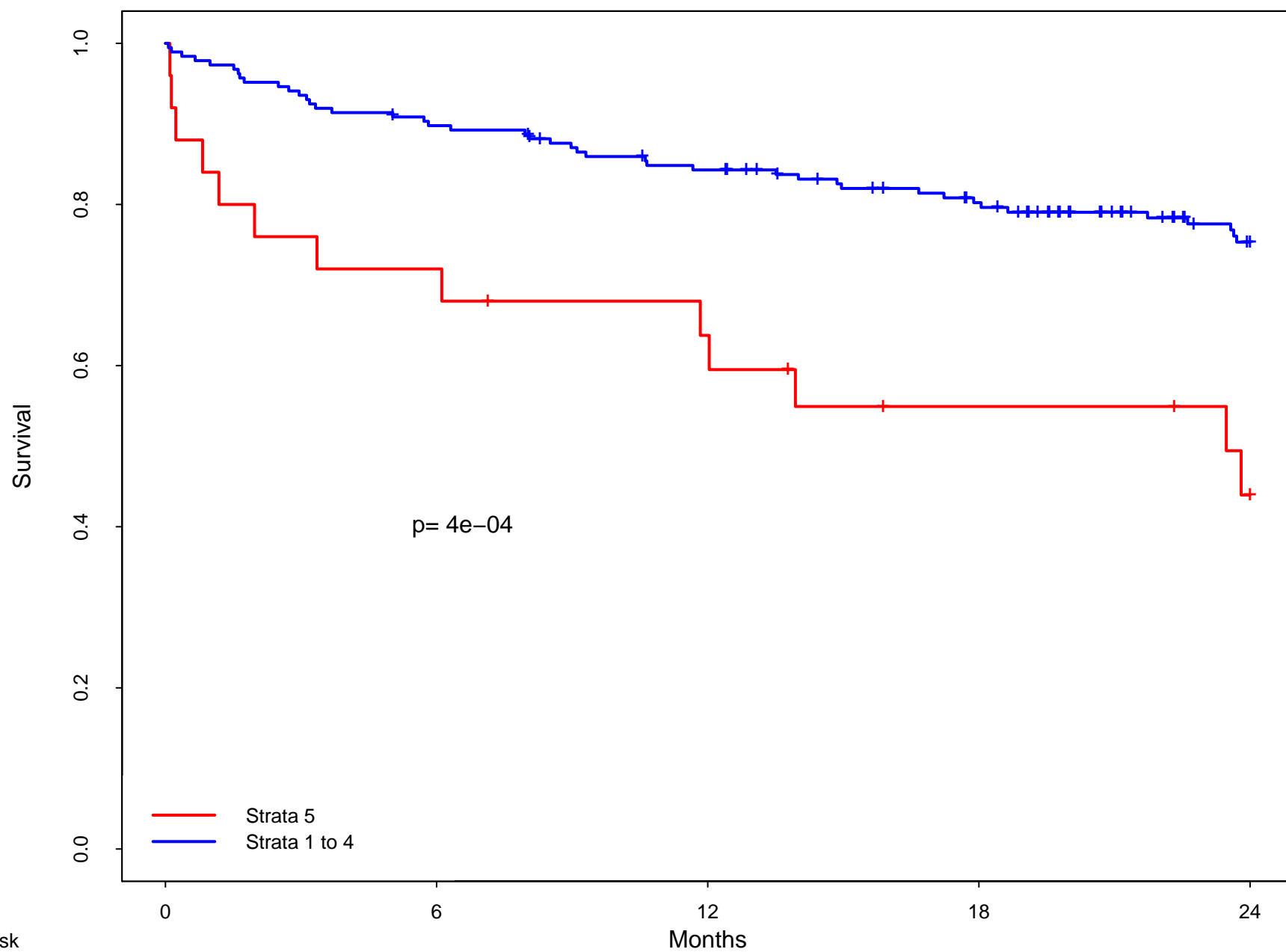

N at risk  
Strata 5  
Strata 1 to 4

25  
186

18  
166

15  
152

11  
135

8  
99

Supplement: Supplementary file 2 — Additional file 2: Supplementary figure 1. Kaplan-Meier-Estimates of survival in patients with (blue line) and without (red line) renal improvement in propensity stratum 1. Supplementary figure 2. Kaplan-Meier-Estimates of survival in patients with (blue line) and without (red line) renal improvement in propensity stratum 2. Supplementary figure 3. Kaplan-Meier-Estimates of survival in patients with (blue line) and without (red line) renal improvement in propensity stratum 3. Supplementary figure 4. Kaplan-Meier-Estimates of survival in patients with (blue line) and without (red line) renal improvement in propensity stratum 4. Supplementary figure 5. Kaplan-Meier-Estimates of survival in patients in propensity strata 1 to 4 (blue line) and in propensity stratum 5 (red line) among the subgroup of patients without renal improvement. Supplementary figure 6. Kaplan-Meier-Estimates of survival in patients in propensity strata 1 to 4 (blue line) and in propensity stratum 5 (red line) among the subgroup of patients with renal improvement. Supplementary figure 7. Kaplan-Meier-Estimates of survival in patients with (dashed lines) and without (solid lines) renal improvement, separately for all 5 propensity strata (stratum 1: black, stratum 2: green, stratum 3: blue, stratum 4: grey, stratum 5: red). Supplementary figure 8. Kaplan-Meier-Estimates of survival in patients with (blue line) and without (red line) renal improvement among the subgroup of patients with NYHA II. Supplementary figure 9. Kaplan-Meier-Estimates of survival in patients with (blue line) and without (red line) renal improvement among the subgroup of patients with NYHA III. Supplementary figure 10. Kaplan-Meier-Estimates of survival in patients with (blue line) and without (red line) renal improvement among the subgroup of patients with baseline NT-proBNP values in the first to third quartile. [file 12882_2021_2274_MOESM2_ESM.zip › Supp5_strata1to4_vs_5inRI0R3.pdf]

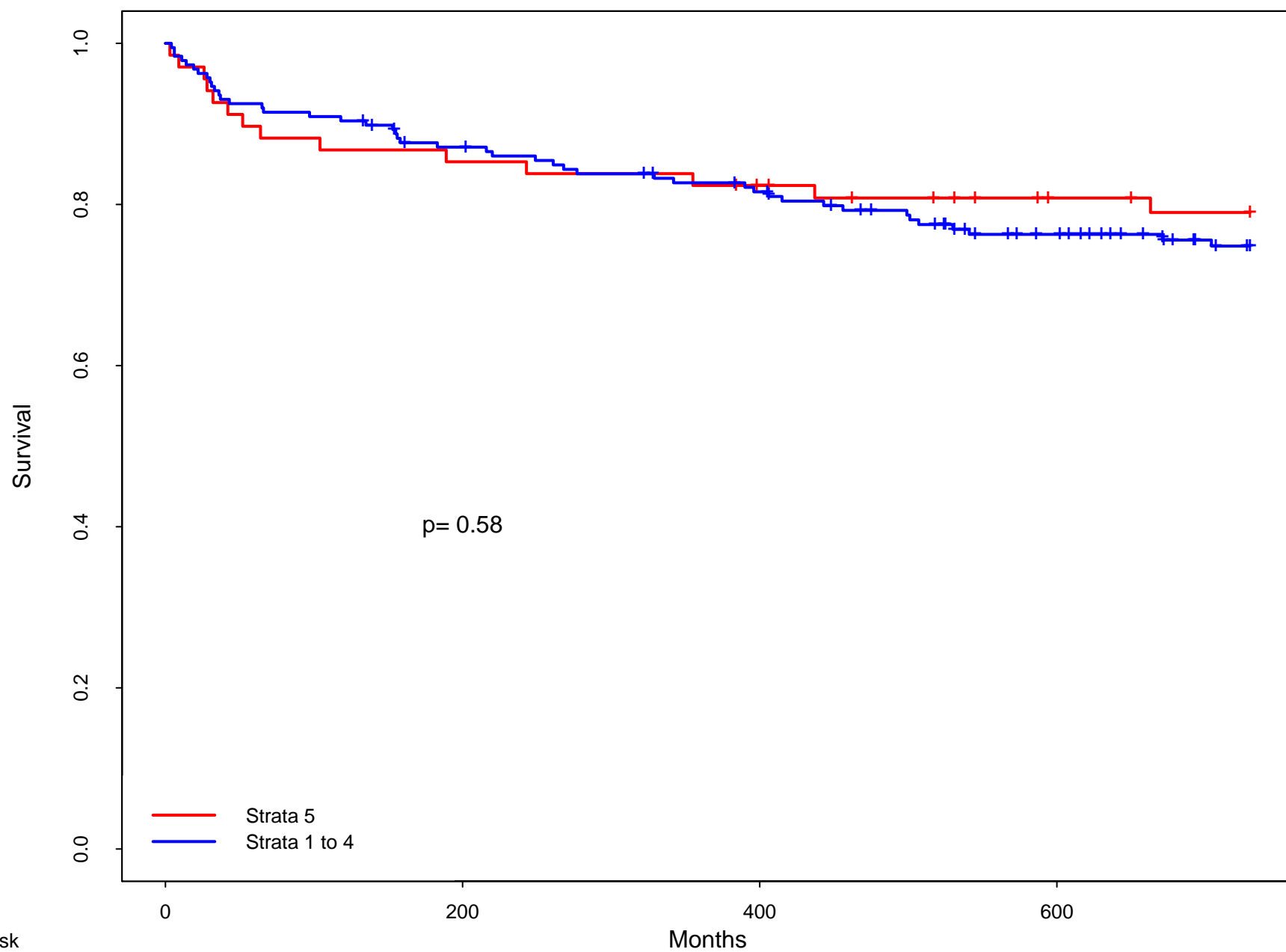

N at risk

|               |     |     |     |     |
|---------------|-----|-----|-----|-----|
| Strata 5      | 68  | 58  | 54  | 46  |
| Strata 1 to 4 | 187 | 159 | 145 | 118 |

Supplement: Supplementary file 2 — Additional file 2: Supplementary figure 1. Kaplan-Meier-Estimates of survival in patients with (blue line) and without (red line) renal improvement in propensity stratum 1. Supplementary figure 2. Kaplan-Meier-Estimates of survival in patients with (blue line) and without (red line) renal improvement in propensity stratum 2. Supplementary figure 3. Kaplan-Meier-Estimates of survival in patients with (blue line) and without (red line) renal improvement in propensity stratum 3. Supplementary figure 4. Kaplan-Meier-Estimates of survival in patients with (blue line) and without (red line) renal improvement in propensity stratum 4. Supplementary figure 5. Kaplan-Meier-Estimates of survival in patients in propensity strata 1 to 4 (blue line) and in propensity stratum 5 (red line) among the subgroup of patients without renal improvement. Supplementary figure 6. Kaplan-Meier-Estimates of survival in patients in propensity strata 1 to 4 (blue line) and in propensity stratum 5 (red line) among the subgroup of patients with renal improvement. Supplementary figure 7. Kaplan-Meier-Estimates of survival in patients with (dashed lines) and without (solid lines) renal improvement, separately for all 5 propensity strata (stratum 1: black, stratum 2: green, stratum 3: blue, stratum 4: grey, stratum 5: red). Supplementary figure 8. Kaplan-Meier-Estimates of survival in patients with (blue line) and without (red line) renal improvement among the subgroup of patients with NYHA II. Supplementary figure 9. Kaplan-Meier-Estimates of survival in patients with (blue line) and without (red line) renal improvement among the subgroup of patients with NYHA III. Supplementary figure 10. Kaplan-Meier-Estimates of survival in patients with (blue line) and without (red line) renal improvement among the subgroup of patients with baseline NT-proBNP values in the first to third quartile. [file 12882_2021_2274_MOESM2_ESM.zip › Supp6_strata1to4_vs_5inRI1R3.pdf]

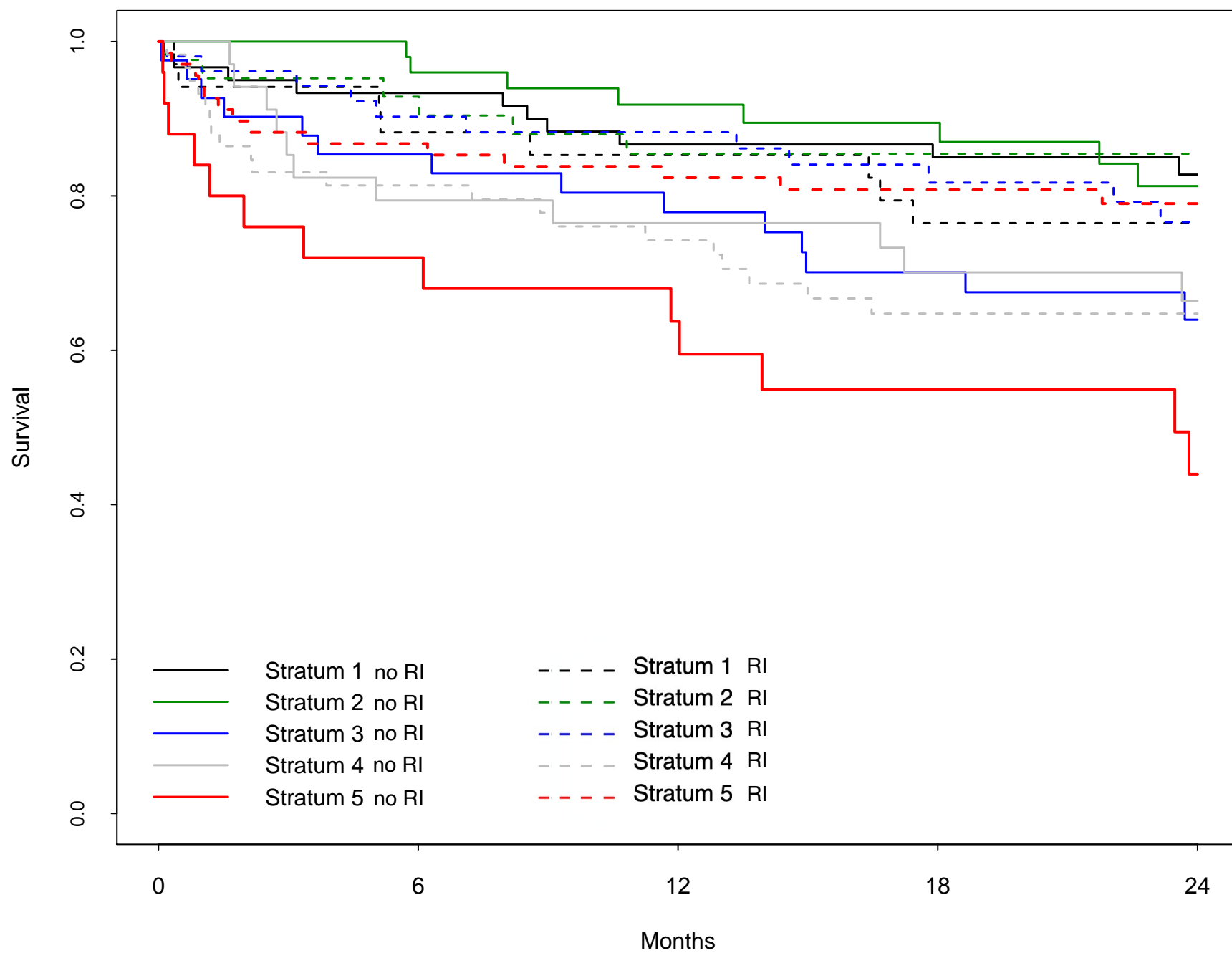

Supplement: Supplementary file 2 — Additional file 2: Supplementary figure 1. Kaplan-Meier-Estimates of survival in patients with (blue line) and without (red line) renal improvement in propensity stratum 1. Supplementary figure 2. Kaplan-Meier-Estimates of survival in patients with (blue line) and without (red line) renal improvement in propensity stratum 2. Supplementary figure 3. Kaplan-Meier-Estimates of survival in patients with (blue line) and without (red line) renal improvement in propensity stratum 3. Supplementary figure 4. Kaplan-Meier-Estimates of survival in patients with (blue line) and without (red line) renal improvement in propensity stratum 4. Supplementary figure 5. Kaplan-Meier-Estimates of survival in patients in propensity strata 1 to 4 (blue line) and in propensity stratum 5 (red line) among the subgroup of patients without renal improvement. Supplementary figure 6. Kaplan-Meier-Estimates of survival in patients in propensity strata 1 to 4 (blue line) and in propensity stratum 5 (red line) among the subgroup of patients with renal improvement. Supplementary figure 7. Kaplan-Meier-Estimates of survival in patients with (dashed lines) and without (solid lines) renal improvement, separately for all 5 propensity strata (stratum 1: black, stratum 2: green, stratum 3: blue, stratum 4: grey, stratum 5: red). Supplementary figure 8. Kaplan-Meier-Estimates of survival in patients with (blue line) and without (red line) renal improvement among the subgroup of patients with NYHA II. Supplementary figure 9. Kaplan-Meier-Estimates of survival in patients with (blue line) and without (red line) renal improvement among the subgroup of patients with NYHA III. Supplementary figure 10. Kaplan-Meier-Estimates of survival in patients with (blue line) and without (red line) renal improvement among the subgroup of patients with baseline NT-proBNP values in the first to third quartile. [file 12882_2021_2274_MOESM2_ESM.zip › Supp7_Surv_RI_Strata1to5 revisedR3.pdf]

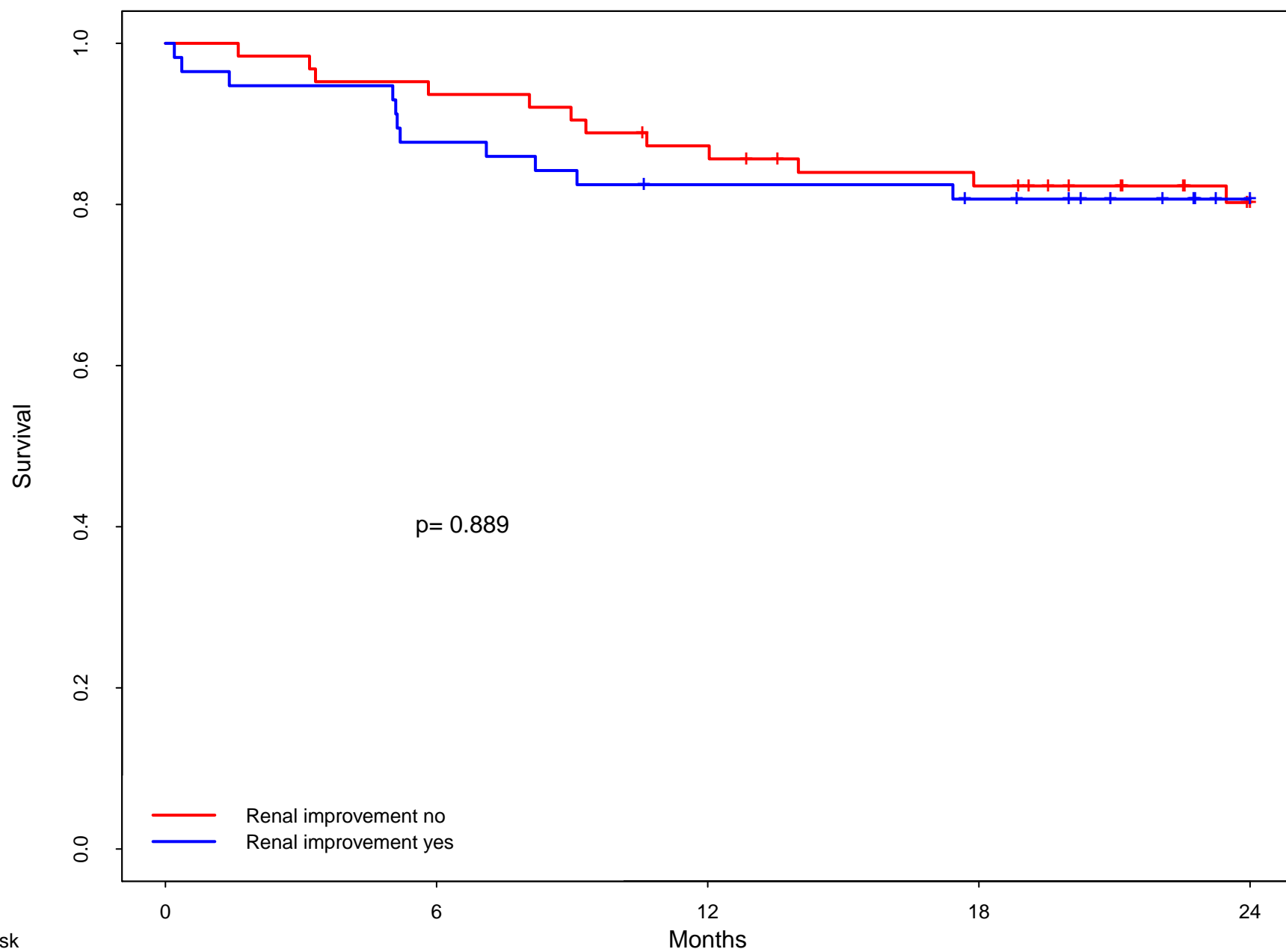

$p=0.889$

Renal improvement no  
Renal improvement yes

N at risk

|    |    |    |    |    |
|----|----|----|----|----|
| 0  | 6  | 12 | 18 | 24 |
| 63 | 59 | 54 | 49 | 38 |
| 57 | 50 | 46 | 44 | 35 |

Supplement: Supplementary file 2 — Additional file 2: Supplementary figure 1. Kaplan-Meier-Estimates of survival in patients with (blue line) and without (red line) renal improvement in propensity stratum 1. Supplementary figure 2. Kaplan-Meier-Estimates of survival in patients with (blue line) and without (red line) renal improvement in propensity stratum 2. Supplementary figure 3. Kaplan-Meier-Estimates of survival in patients with (blue line) and without (red line) renal improvement in propensity stratum 3. Supplementary figure 4. Kaplan-Meier-Estimates of survival in patients with (blue line) and without (red line) renal improvement in propensity stratum 4. Supplementary figure 5. Kaplan-Meier-Estimates of survival in patients in propensity strata 1 to 4 (blue line) and in propensity stratum 5 (red line) among the subgroup of patients without renal improvement. Supplementary figure 6. Kaplan-Meier-Estimates of survival in patients in propensity strata 1 to 4 (blue line) and in propensity stratum 5 (red line) among the subgroup of patients with renal improvement. Supplementary figure 7. Kaplan-Meier-Estimates of survival in patients with (dashed lines) and without (solid lines) renal improvement, separately for all 5 propensity strata (stratum 1: black, stratum 2: green, stratum 3: blue, stratum 4: grey, stratum 5: red). Supplementary figure 8. Kaplan-Meier-Estimates of survival in patients with (blue line) and without (red line) renal improvement among the subgroup of patients with NYHA II. Supplementary figure 9. Kaplan-Meier-Estimates of survival in patients with (blue line) and without (red line) renal improvement among the subgroup of patients with NYHA III. Supplementary figure 10. Kaplan-Meier-Estimates of survival in patients with (blue line) and without (red line) renal improvement among the subgroup of patients with baseline NT-proBNP values in the first to third quartile. [file 12882_2021_2274_MOESM2_ESM.zip › Supp8_Surv_RI_in_NYHA2R3.pdf]

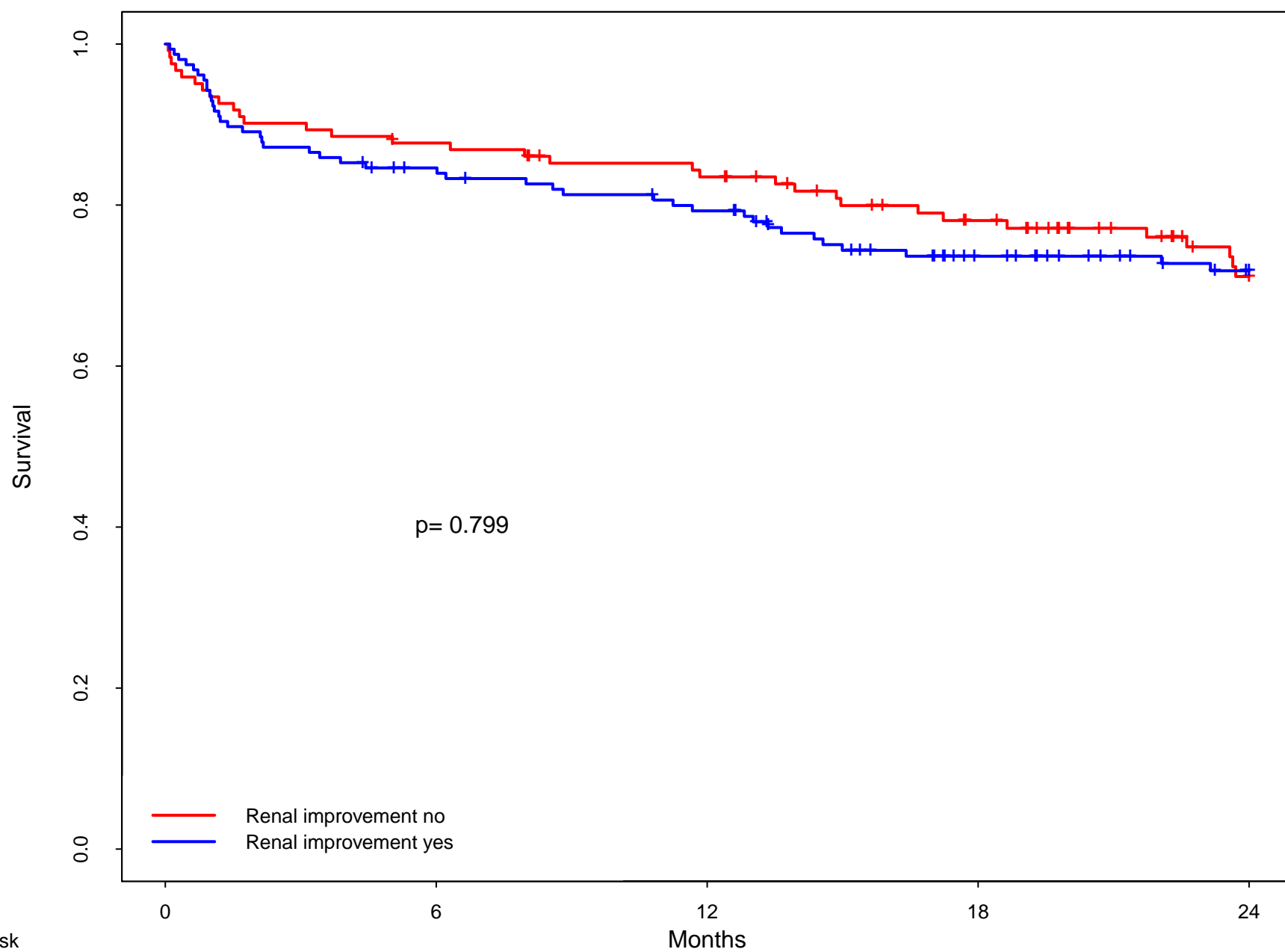

N at risk

|                       | 0   | 6   | 12  | 18 | 24 |
|-----------------------|-----|-----|-----|----|----|
| Renal improvement no  | 122 | 106 | 98  | 82 | 58 |
| Renal improvement yes | 156 | 128 | 118 | 93 | 76 |

Supplement: Supplementary file 2 — Additional file 2: Supplementary figure 1. Kaplan-Meier-Estimates of survival in patients with (blue line) and without (red line) renal improvement in propensity stratum 1. Supplementary figure 2. Kaplan-Meier-Estimates of survival in patients with (blue line) and without (red line) renal improvement in propensity stratum 2. Supplementary figure 3. Kaplan-Meier-Estimates of survival in patients with (blue line) and without (red line) renal improvement in propensity stratum 3. Supplementary figure 4. Kaplan-Meier-Estimates of survival in patients with (blue line) and without (red line) renal improvement in propensity stratum 4. Supplementary figure 5. Kaplan-Meier-Estimates of survival in patients in propensity strata 1 to 4 (blue line) and in propensity stratum 5 (red line) among the subgroup of patients without renal improvement. Supplementary figure 6. Kaplan-Meier-Estimates of survival in patients in propensity strata 1 to 4 (blue line) and in propensity stratum 5 (red line) among the subgroup of patients with renal improvement. Supplementary figure 7. Kaplan-Meier-Estimates of survival in patients with (dashed lines) and without (solid lines) renal improvement, separately for all 5 propensity strata (stratum 1: black, stratum 2: green, stratum 3: blue, stratum 4: grey, stratum 5: red). Supplementary figure 8. Kaplan-Meier-Estimates of survival in patients with (blue line) and without (red line) renal improvement among the subgroup of patients with NYHA II. Supplementary figure 9. Kaplan-Meier-Estimates of survival in patients with (blue line) and without (red line) renal improvement among the subgroup of patients with NYHA III. Supplementary figure 10. Kaplan-Meier-Estimates of survival in patients with (blue line) and without (red line) renal improvement among the subgroup of patients with baseline NT-proBNP values in the first to third quartile. [file 12882_2021_2274_MOESM2_ESM.zip › Supp9_Surv_RI_in_NHYA3R3.pdf]
